# Survey of local cannabidiol use in parents of children with epilepsy in Thailand: the prevalence, perceptions, and knowledge

**DOI:** 10.1186/s42238-022-00155-8

**Published:** 2022-07-26

**Authors:** Monsicha Ngampoopun, Charcrin Nabangchang, Piradee Suwanpakdee

**Affiliations:** Neurology Division, Department of Pediatrics, Phramongkutklao Hospital, Bangkok, Thailand

**Keywords:** Cannabidiol, Epilepsy, Thailand, Pediatrics

## Abstract

**Background:**

In 2019, Thailand legalized cannabidiol (CBD) for intractable epilepsy. The purpose of this study was to collect information regarding the experience and knowledge of CBD use in pediatric epilepsy. To the best of our knowledge, this is the first CBD survey in pediatric epilepsy in Southeast Asia.

**Method:**

We performed a cross-sectional survey among all parents of pediatric epilepsy patients seen in the Pediatric Neurology Clinic at Phramongkutklao Hospital, Bangkok, Thailand between November 2018 and July 2020. The survey comprised 34 questions that assessed the demographics, knowledge, experiences, and opinions of parents/guardians regarding CBD use. The results were summarized using descriptive statistics. In addition, logistic regression was used to predict the factors for CBD use.

**Results:**

Overall, 166 respondents (100%) participated in the study. Among the respondents, 9% have experienced using CBD; 56.25% of these reported reduced seizure frequency. CBD products were mostly obtained from folk healers (31.25%) and foreign products (25%). Common adverse effects included headache and nausea (31.5%). The number of anti-seizure medications (OR: 12.28, 95% CI: 1.27–118.8), knowledge of CBD as treatment for epilepsy (OR: 14.7, 95% CI: 1.43–150.87), and knowledge of CBD side effects (OR: 12.73, 95% CI: 2.77–58.43) were factors significantly associated with CBD use. Regarding CBD knowledge, our survey showed 80.72% of the respondents did not know the CBD compound for treating epilepsy, and 89.16% were not aware of CBD side effects. Interestingly, despite a lack of knowledge, 77.11% of the respondents expressed willingness to participate in future CBD trials.

**Conclusion:**

Our survey highlights that half of the parents of patients who previously used CBD reported reduced seizure frequency; however, none became seizure-free. Additionally, there were gaps in knowledge regarding the use of CBD. These findings suggest that the implementation of cannabidiol knowledge is crucial for both public and healthcare professionals. Survey limitations due to the retrospective nature of the self-report could have resulted in recall bias.

## Background

Despite the development of new anti-seizure medications (ASMs), 30% of pediatric epilepsy patients remain refractory to treatment; these cases are considered as having drug-resistant or intractable epilepsy (Berg et al. 2001; Kwan et al. 2010). The burden of intractable epilepsy on children and their families significantly affects their development and quality of life. The use of multiple ASMs is associated with increased risks of adverse drug effects and drug interactions, poorer compliance, and greater expenses (Atugonza et al. 2016). These factors draw patients towards alternative therapeutic interventions and even untested treatment options such as cannabidiol (CBD) products. A previous study demonstrated that CBD has a therapeutic anti-seizure effect in many animal models (Jones et al. 2012). Moreover, a randomized controlled trial demonstrated that CBD significantly reduced the frequency of seizures in children with Dravet syndrome, Lennox–Gastaut syndrome, and other forms of intractable epilepsy (Chu Sin Chung and Kieffer 2013; Ligresti et al. 2016; Nazıroğlu 2015). A recent systematic review identified six randomized controlled studies that reported CBD at a dose of 20 mg/kg/day was more effective than placebo in achieving complete seizure freedom (Stockings et al. 2018). In terms of safety, the adverse effects of CBD are mild and only observed during the first month of use (Silva et al. 2020).

Thailand has a long relationship with cannabis as the plant appears to have been introduced to the country from India. In a 2001 report, the Drug Enforcement Administration’s Intelligence Division revealed that Thailand was Southeast Asia’s major cultivator of CBD and producer of marijuana in the 1970s and 1980s, both as a medicine and as a recreational drug (Sawasdee Clinic 2019). However, the use of CBD as medication in Thailand was previously outlawed; in the absence of physician supervision, patients used it to treat seizures due to a lack of legal products.

In February 2019, Thailand became the first Asia-Pacific country that legalized CBD, with South Korea following in March 2019 (Gan 2019). The goal of legalization was to expand treatment options for patients with epilepsy, chronic pain, and other medical conditions. Under the new law, Thai people with approved health conditions can use CBD after obtaining a prescription from a certified physician. The permissible indications for therapy with CBD are intractable epilepsy, severe vomiting from chemotherapy, multiple sclerosis, and intractable neuropathic pain (Department of medical services 2020). The supply and distribution is operated by a nationwide network of marijuana medical clinics under the Ministry of Public Health. Recently, in August 2020, the National Health Security Office (NHSO) covered the cost of CBD expenses for specific medical conditions to assist low-income patients.

Despite the increasing popularity of CBD use, evidence regarding CBD for pediatric epilepsy in Thailand is scarce. Therefore, this study aimed to obtain information regarding the experience of parents/guardians in treating epilepsy in their children using CBD as well as the patients’ knowledge and understanding of CBD.

## Methods

The study was conducted as a cross-sectional survey between 2018 and 2020 with a validated questionnaire comprising 34 questions that assessed the demographics, clinical factors, seizure types, knowledge, experiences, and opinions of parents/guardians regarding CBD use in pediatric epilepsy. The questionnaire was translated from the Australian Survey (Suraev et al. 2017) and the Parent Survey Report (Porter and Jacobson 2013) to Thai by a linguist. To determine whether the respondents will be able to understand the questions, the questionnaire was validated by 20 multi-disciplinary personnel, including staff, patient’s relatives, and nursing assistants. The study design was approved by the Institutional Review Board of the Royal Thai Army Medical Department, and all respondents gave their written consent. The study data and informed consents were collected from only one parent per pediatric epilepsy patient who visited the pediatric neurology clinic at Phramongkutklao Hospital, Bangkok, Thailand. We defined the home of the study respondents as “central” if they resided in Bangkok and “rural” if they resided outside Bangkok.

The survey used reference content from “Guidance on Cannabis for Medical Use”, Department of medical services, Thailand (Department of medical services 2020). The study population was parents/guardian of children aged ≤18 years who were diagnosed with epilepsy.

Data were analyzed using SPSS 19.0 for Mac (IBM Corporation, Armonk, NY). Descriptive statistics, including means, standard deviations, confidence intervals, and simple percentages, were used to describe the demographic data, knowledge, experiences, and reasons for using CBD. Each independent variable was first entered into a univariate binary logistic regression analysis. Variables that predicted cannabis use with a degree of significance of *p* < 0.05 were entered into a multivariate forward conditional binary logistic regression analysis. The covariates used in the multivariate logistic regression analysis evaluated risk factors predicting CBD use.

## Results

### Demographic information

There were 166 respondents (for 166 patients), and many of the respondents were from the central region (66.27%). The demographic information of the respondents is shown in Table 1.

**Table 1:** Demographic and clinical characteristics of 166 pediatric epilepsy patients in Thailand

	***N*** (%)
**Total patients**	166
**Gender**	
Female	76 (45.78)
Male	90 (54.22)
**Age** – median (min-max)	12 (3 months–18 years)
**Seizure onset (month)** – median (min-max)	36 (1–192)
**Seizure type**	
Focal seizure with impair awareness	159 (95.78)
Generalized seizure	7 (4.22)
**Cause of seizure**	
Unknown	94 (56.63)
Remote symptomatic epilepsy	32 (19.28)
Acute symptomatic epilepsy	21 (12.64)
Genetic syndrome	19 (11.45)
**Total previous ASMs** – median (Min–Max)	3 (1–10)
**Patient relationship**	
Parents	139 (83.73)
Cousins/siblings	13 (7.83)
Patients	11 (6.63)
Guardian	3 (1.81)
**Income/month**	
< 50,000 baht	147 (88.56)
≥ 50,000 baht	19 (11.44)

*ASMs* anti-seizure medications

The patients were mostly male (54.22%), and their ages ranged from 3 months to 18 years (median, 12 years). The patients had received a median of two ASMs (range, 0–7) concomitantly.

### Prevalence and use of CBD in pediatric epilepsy

Among the patients, 9.64% (16/166) and 3.61% (6/166) previously used and are currently using CBD, respectively.

Among those who previously used CBD, 56.25% (9/16) reported reduced seizure frequency and ASMs. The main reasons for CBD use were to manage treatment-resistant epilepsy (56.25%, 9/16) and because it was recommended by an acquaintance (37.5%, 6/16). CBD products were obtained from folk healers (31.25%, 5/16) and foreign products (25%, 4/16). Interestingly, 81.25% (13/16) of patients who previously used CBD were not aware of the composition of CBD products.

The most common type of CBD product used in treating epilepsy was CBD oil (81.25%, 13/16) followed by boiled fresh CBD (12.5%, 2/16). The dose of CBD used by patients varied from 1 drop per day to 4 drops twice a day; the milligrams of CBD per milliliter was unknown. The duration of usage was not more than 1 month in 56.25% (9/16) of the patients. Adverse effects of CBD, such as headache and nausea, were noted in 31.5% (5/16) of the patients. Meanwhile, lack of seizure improvement and inability to procure a CBD product were the reasons for discontinuing CBD in 43.75% (7/16) and 6.25% (1/16) of the patients, respectively.

### Knowledge and perception of CBD use

Most parents (71.68%, 119/166) were aware that CBD was legally approved in Thailand; however, only 48.79% (81/166) were aware of CBD as treatment for epilepsy in children. Furthermore, 80.72% (134/166) did not know the composition of CBD required for treating epilepsy, and 89.16% (148/166) were not aware of any side effects of CBD. Regarding knowledge on CBD, 89.76% (149/166) answered that cannabis is a plant that does not absorb heavy metals, pesticides, and toxins from the soil, 39.16% (65/166) answered that CBD could cure cancer, and 56.02% (93/166) answered that CBD could be used in women during pregnancy and breast-feeding (Table 2).

**Table 2:** Knowledge of cannabidiol use among Thai parents of children with epilepsy

	Parents/guardians of children with epilepsy ***N*** = 166 (%)	
Did you know that cannabidiol is used to treat epilepsy in children?	Yes No	81 (48.79) 85 (51.21)
Did you know that cannabidiol is legally approved for therapeutic use in Thailand?	Yes No	119 (71.69) 47 (28.31)
Do you know any side effects you need to be aware of after using cannabidiol products?	Yes No	18 (10.84) 148 (89.16)
Do you know what compound in cannabis should be used to treat epilepsy in children? -THC -CBD -Don’t know		2 (1.21) 30 (18.07) 134 (80.72)
Do you think that cannabis is a plant that absorbs heavy metals, pesticides, and toxins from the soil?	Yes No	149 (89.76) 17 (10.24)
Do you think cannabidiol can cure cancer?	Yes No	101 (60.84) 65 (39.16)
Do you think that cannabidiol should not be used in pregnant or breastfeeding women as it can affect the baby?	Yes No	93 (56.02) 73 (43.98)

*THC* tethahydrocannabinol, *CBD* cannabidiol

Regarding information on who will issue the CBD prescription, 77.71% (129/166) replied with the pediatric neurologist trained by the Department of Medical service. Additionally, some respondents answered that folk healers (4.83%, 8/166) and those practicing Thai traditional medicine (7.83%, 13/166) could prescribe CBD for pediatric epilepsy management.

### Predictors associated with CBD product use

Regarding factors for CBD use, the number of ASMs (OR: 12.28, 95% CI: 1.27–118.8), knowledge of CBD as a treatment for epilepsy (OR: 14.7, 95% CI: 1.43–150.87), and knowledge of CBD side effects (OR: 12.73, 95% CI: 2.77–58.43) were factors significantly associated with CBD use as shown by multivariate analyses (Table 3).

**Table 3:** Crude and adjusted odd ration (OR) and 95% confidence interval from multivariable logistic regression of factors associated with the use of cannabidiol to treat childhood epilepsy

	Used ***n*** (%)	Never use ***n*** (%)	Crude odds ratio (95%CI)	***p***-value	Adjusted odds ratio* (95%CI)	***p***-value
**Sex**						
**Female**	5 (6.58)	71 (93.42)	1		1	
**Male**	11 (12.22)	79 (87.78)	1.98 (0.66–5.97)	0.226	1.35 (0.33–5.53)	0.678
**Age**						
**Age (years), median (Min–Max)**	11 (2–18)	12 (0.25–18)	0.96 (0.87–1.06)	0.411	0.88 (0.77–1.02)	0.083
**Number previous ASMs**						
**<3**	1 (1.39)	71 (98.61)	1		1	
**3+**	15 (15.96)	79 (84.04)	13.48 (1.74–104.66)	0.013	12.28 (1.27–118.8)	**0.03**
**Median (Min–Max)**	4 (1–6)	2 (0–7)				
**Income/month**						
**0–50,000 baht**	12 (8.16)	135 (91.84)	1		1	
**≥ 50,000 baht**	4 (21.05)	15 (78.95)	3 (0.86–10.48)	0.085	1.67 (0.29–9.54)	0.562
**Domicile**						
**Central region**	10 (9.09)	100 (90.91)	1		1	
**Rural area**	6 (10.71)	50 (89.29)	1.2 (0.41–3.49)	0.738	0.66 (0.16–2.7)	0.565
**Knowing cannabidiol in epilepsy**						
**Yes**	15 (18.52)	66 (81.48)	19.09 (2.46–148.26)	0.005	14.7 (1.43–150.87)	**0.024**
**No**	1 (1.18)	84 (98.82)	1		1	
**Knowing cannabidiol side effects**						
**Yes**	8 (44.44)	10 (55.56)	14 (4.34–45.17)	<0.001	12.73 (2.77–58.43)	**0.001**
**No**	8 (5.41)	140 (94.59)	1		1	

*ASMs* antiseizure medications

*Adjusted for sex, child’s age, number of previous ASMs, income, domicile, knowing cannabidiol in epilepsy, and knowing cannabidiol side effects

### Reasons in favor and against the use of CBD products for treating epilepsy

Among epilepsy patients, 56.25% (9/16) were reported to have used CBD products due to treatment-resistant epilepsy, and 37.5% (6/16) used CBD as it was recommended by an acquaintance. Meanwhile, 56% (84/150) of children with no history of using CBD products for epilepsy reported the lack of medical advice and support from medical doctors as the leading cause for not trying CBD products, while 18.67% (28/150) were concerned regarding their safety. The reasons for using CBD products to manage epilepsy in children are summarized in Table 4.

**Table 4:** Reasons for using and against using cannabidiol products to manage epilepsy

**Reasons for using of CBD products for epilepsy**	***N = 16 (%)***
-Treatment-resistant epilepsy	9 (56.25)
-Unacceptable ASMs side-effects	2 (12.50)
-Success stories from media	3 (18.75)
-To manage other health conditions (and epilepsy)	2 (12.50)
-Recommended by acquaintances	6 (37.50)
-Want to try	5 (31.25)
**Reasons against or hesitation towards the use of CBD products for epilepsy**	***N = 150 (%)***
-Need for medical supervision	84 (56)
-Concerns over safety	28 (18.67)
-Difficulties with sourcing reliable supply	7 (4.67)
-Epilepsy currently controlled	20 (13.33)
-Illegal status	11 (7.33)

*ASMs* antiseizure medications, *CBD* cannabidiol

### Future CBD trials

Among the parents/guardians of epilepsy patients, 77.11% (128/166) expressed a willingness to participate in future CBD clinical trials, with the most common reason being the desire to cure epilepsy. Additionally, 72.89% (121/166) were uncertain whether CBD was a cure for epilepsy, while 25.9% (43/166) believed it could improve seizures. Lastly, the acceptable costs for CBD in treating pediatric epilepsy were <1000 baht (32.42 US dollars)/month in 42.77% (71/166), and 30.12% (50/166) were inconvenienced by the cost of the treatment.

## Discussion

This study aimed to obtain information on the experience in treating epilepsy in children using CBD as well as knowledge and understanding of CBD. To our knowledge, this is the first CBD survey on pediatric epilepsy in Southeast Asia.

In a previous Australian survey (Suraev et al. 2017), the prevalence of CBD use for pediatric epilepsy was 13%, the main reason for using CBD was treatment-resistant epilepsy, and the main factor for CBD use was the number of ASMs; all of which are consistent with our results. Other factors for CBD use in the Australian survey were the use of a ketogenic diet, focal impaired awareness seizures, and unknown seizure types. In our study, there was no association between gender, age, and socioeconomic status with CBD use.

Regarding the efficacy of CBD in epilepsy, Porter et al. (Porter and Jacobson 2013) reported a parent survey of CBD used in 19 pediatric treatment-resistant epilepsy patients and found that 84% (16/19) of the parents reported a reduction in their child’s seizure frequency. Two parents reported that their child became seizure-free, and 63% (12/19) of parents weaned their child from other ASMs following CBD-enriched CBD treatment. Compared to our study, parents/guardians reported seizure reduction in 56.25% of patients; however, none became seizure-free.

The side effects in patients were then analyzed. Porter et al. reported improved cognition, mood, and sleep after CBD administration (Porter and Jacobson 2013), which were rarely reported with ASMs (Bourgeois 2013). Devinsky et al. studied 214 patients with epilepsy aged 1–30 years who received oral CBD at 2–5 mg/kg per day and demonstrated that the common side effects (10%) were somnolence, decreased appetite, diarrhea, and fatigue (Devinsky et al. 2016). Compared to our survey, the most common side effects were nausea and headache in 31.5% of patients, and no beneficial side effects were reported.

In terms of knowledge, an Australian nationwide survey indicated that both parents/guardians of children and adults who have used cannabis extracts for epilepsy reported a high level of perceived efficacy with cannabis products (Suraev et al. 2017). In contrast, our survey showed that half of the parents were not aware of CBD being used as treatment for epilepsy in children, and two-thirds were unaware of the composition of CBD required for treating epilepsy as well as its side effects. Interestingly, despite an apparent lack of knowledge on CBD, most parents (77.11%) favored the legalization of CBD trials. Regardless, our questionnaire was unable to demonstrate the association between parent characteristics and their knowledge about CBD treatment for epilepsy. We hypothesized that one of the important factors for the lack of knowledge was the insufficient guidance by healthcare professionals.

A previous systematic review of healthcare professionals’ beliefs, knowledge, and concerns regarding CBD use reported that healthcare professionals lacked confidence and self-reporting competence as well as harbored concerns regarding the associated risks of CBD (Gardiner et al. 2019). Elliott et al. reported diverse opinions and experiences of neurologists in Canada on the use of CBD for the treatment of drug-resistant epilepsy in children. Most neurologists interviewed expressed that CBD is a variable option; however, important gaps in evidence-based knowledge were identified, including limited knowledge about the medical properties of cannabinoids beyond CBD (Elliott et al. 2020). To date, although no survey regarding healthcare professionals’ and CBD exists in Thailand, parents in our study who did not use CBD for epilepsy treatment mentioned the lack of medical advice as a reason. This highlights that physicians should impart more knowledge on the therapeutic use of CBD, medication adjustments, dosage, and possible side effects to provide valuable information to patients during consultations for alternative treatments.

We recognize that our study has multiple limitations, including the retrospective nature of the parents/guardians’ self-report, which could have resulted in poor recollection and recall bias. Additionally, this study was conducted in a single clinical setting; thus, a prospective multicenter study is necessary. Moreover,

the patients in whom seizure frequency was reduced used CBD without medical advice from a physician, resulting in the varied doses, chemical ingredients, and duration of CBD use. Hence, we cannot compare the dose and duration of CBD use in this study with other previous clinical trial studies. Despite the limitations of our study, we believe that the data we have reported here are relevant given the paucity of prospective clinical trial studies in this population of pediatric patients with epilepsy.

## Conclusions

Our survey revealed that half of the parents of patients who previously used CBD reported reduced seizure frequency; however, no patient became seizure-free. In addition, although Thailand legalized CBD in 2019, we found significant gaps in the knowledge of parents and guardians regarding CBD use in Thailand. These findings suggest that imparting knowledge and disseminating information regarding CBD is crucial before initiating CBD use. Moreover, further evidence-based studies on CBD are warranted.

## Data Availability

The dataset analyzed during the current study is available from the corresponding author to researchers on reasonable request.

## Abbreviations

ASMs: Anti-seizure medications
CBD: Cannabidiol
